# The Extended Functional Neuroanatomy of Emotional Processing Biases for Masked Faces in Major Depressive Disorder

**DOI:** 10.1371/journal.pone.0046439

**Published:** 2012-10-08

**Authors:** Teresa A. Victor, Maura L. Furey, Stephen J. Fromm, Patrick S. F. Bellgowan, Arne Öhman, Wayne C. Drevets

**Affiliations:** 1 National Institutes of Health, National Institute of Mental Health, Bethesda, Maryland, United States of America; 2 Laureate Institute for Brain Research, Tulsa, Oklahoma, United States of America; 3 Department of Clinical Neuroscience, Karolinska Institutet, Stockholm, Sweden; 4 Faculty of Community Medicine, University of Tulsa, Tulsa, Oklahoma, United States of America; The University of Melbourne, Australia

## Abstract

**Background:**

Major depressive disorder (MDD) is associated with a mood-congruent processing bias in the amygdala toward face stimuli portraying sad expressions that is evident even when such stimuli are presented below the level of conscious awareness. The extended functional anatomical network that maintains this response bias has not been established, however.

**Aims:**

To identify neural network differences in the hemodynamic response to implicitly presented facial expressions between depressed and healthy control participants.

**Method:**

Unmedicated-depressed participants with MDD (n = 22) and healthy controls (HC; n = 25) underwent functional MRI as they viewed face stimuli showing sad, happy or neutral face expressions, presented using a backward masking design. The blood-oxygen-level dependent (BOLD) signal was measured to identify regions where the hemodynamic response to the emotionally valenced stimuli differed between groups.

**Results:**

The MDD subjects showed greater BOLD responses than the controls to masked-sad versus masked-happy faces in the hippocampus, amygdala and anterior inferotemporal cortex. While viewing both masked-sad and masked-happy faces relative to masked-neutral faces, the depressed subjects showed greater hemodynamic responses than the controls in a network that included the medial and orbital prefrontal cortices and anterior temporal cortex.

**Conclusions:**

Depressed and healthy participants showed distinct hemodynamic responses to masked-sad and masked-happy faces in neural circuits known to support the processing of emotionally valenced stimuli and to integrate the sensory and visceromotor aspects of emotional behavior. Altered function within these networks in MDD may establish and maintain illness-associated differences in the salience of sensory/social stimuli, such that attention is biased toward negative and away from positive stimuli.

## Introduction

A mood-congruent emotional processing bias toward negative information is a robust finding in major depressive disorder (MDD). Neuropsychological studies consistently have reported that depressed subjects show enhanced attention and memory for emotionally negative as compared to positive or neutral stimuli [1]–[7]. Similarly, functional neuroimaging studies report exaggerated neurophysiological activity in the amygdala and other structures in response to negative stimuli, including sad faces or words, in depressed subjects versus healthy controls [8]–[15].

In a recent functional MRI (fMRI) study of implicit emotional processing biases in depressed and healthy participants [8] we reported that the negative bias in MDD is evinced by the hemodynamic responses of the amygdala to emotionally expressive face stimuli presented below conscious awareness using a backward masking technique. Specifically, the amygdala response was greater to masked-*sad* faces versus masked-*happy* or masked-*neutral* faces in unmedicated depressed subjects, but was greater toward masked-*happy* faces versus masked-*sad* or masked-*neutral* faces in healthy controls (see also [16], [17]).

The amygdala plays a pivotal role in evaluating the behavioral salience of sensory stimuli, partly through subcortical networks that rapidly respond to stimulus features and objects processed at both conscious and non-conscious levels [18]–[20]. In this role the amygdala forms part of extended anatomical networks that involve other mesiotemporal lobe structures, the sensory thalamus, the primary and associative sensory cortices, and the medial and orbital prefrontal cortex. Together, these regions function to modulate the neural and behavioral responses to emotional stimuli on the basis of learning, context and changing reinforcement contingencies [21]–[24]. The current study aimed to elucidate the extended functional anatomical network that participates with the amygdala in maintaining emotional processing biases in MDD, by identifying other brain regions where the hemodynamic response to masked-sad versus masked-happy face stimuli differed between depressed and healthy control participants.

## Methods

### Ethics Statement

The research was conducted at the National Institute of Mental Health Intramural Research Program under Protocol 04-M-0002: “The Functional Neuroanatomy of Emotion Regulation in Major Depressive Disorder (MDD)", which received initial NIMH IRB approval on August 19, 2003 with subsequent approval at each annual continuing review. The most recent Continuing Renewal application for this protocol was approved by the NIMH Combined Neuroscience (CNS) Institutional Review Board (IRB) on August 4, 2009.

### Participants

The study samples were the same as those characterized in our initial report of data primarily limited to the amygdala [8], and were composed of 22 unmedicated adults who met the Diagnostic and Statistical Manual of Mental Disorders-IV-TR [25] criteria for having MDD in a current major depressive episode (MDD) and 25 healthy controls (HC). Briefly, right-handed individuals [26], ages 18 to 50 years were recruited from the Washington, D.C. metropolitan area. Prior to enrolling in the study, each person underwent a screening evaluation including a medical and psychiatric history, laboratory testing that included a urine drug screen, and neuromorphological MRI. The psychiatric diagnosis was established by a semi-structured interview with a psychiatrist and the Structured Clinical Interview for DSM-IV-TR (SCID) [27]. A family history of psychiatric disorders was evaluated by The Family Interview for Genetic Studies [28].

Subjects were excluded from participation if they manifested any of the following conditions: 1) serious suicidal ideation or behavior, 2) major medical or neurological disorders, 3) exposure within three weeks (eight weeks for fluoxetine) to psychotropic drugs or to other medications likely to affect cerebral blood flow or cognitive function, 4) history of drug or alcohol abuse within the past one-year or a lifetime history of drug or alcohol dependence [27], excepting nicotine, 5) current pregnancy or breastfeeding, or 6) general MRI exclusions. The healthy volunteers were excluded if they had a history of any major psychiatric disorder or a first-degree relative with a mood or anxiety disorder. Each participant received a thorough explanation of the study, provided written informed consent as approved by the National Institute of Health Combined Neuroscience Institutional Review Board, and received financial compensation for their involvement in the study.

Intelligence testing and depressive symptom ratings were performed prior to scanning. Assessments included the Wechsler Abbreviated Scale of Intelligence (WASI) [29], the Hamilton Depression Rating Scale (HAM-D, 24-item) [30], the Automatic Thoughts Questionnaire (ATQ) [31], the Inventory of Depressive Symptomatology: Self-Rating (IDS-SR) [32], the State-Trait Anxiety Inventory (STAI) [33] and the Thought Control Questionnaire (TCQ) [34].

### fMRI Backward Masking Task

Participants were scanned while performing a backward masking task [8]. A complete description of the backward masking task design was included in our initial report [8]. To summarize, participants were shown two neutral target faces before the start of each of four ten-minute runs. During the task they responded to the presence of a target face by pressing the top button on a two-button response box or a non-target face by pressing the bottom button on a response box. The stimuli displayed sad, happy or neutral faces, however, the participants were asked to respond to the faces as target or non-target based on their identity, irrespective of the emotional expression. Each task-trial consisted of an emotional stimulus pair of faces including either sad-neutral (SN), happy-neutral (HN), neutral-sad (NS), neutral-happy (NH) or neutral-neutral (NN) faces. The first face was displayed for 26 ms, followed by a 2^nd^ “masking" face for 107 ms to inhibit conscious perception of the first face. The current paper describes the data limited to trials in which the emotional face stimuli were presented in the masked position. The SN, HN, NS, and NH stimulus types each were shown eight times and the NN type was presented 16 times during each of four runs in a pseudo-randomized, mixed-trial design. Within a single trial, the *identity* of the masked face was never the same as the identity of the masking face, but the gender of the two face stimuli was always the same. A 10 to 13 second inter-stimulus interval followed each stimulus pair to allow the hemodynamic response to return to baseline prior to the next presentation.

Stimuli were presented to subjects in the scanner gantry using E-Prime software (Psychological Software Tools, Pittsburgh, PA) and a cloned display projection on a Monarch Hornet PC computer with a cathode ray tube monitor at 75 Hz to enable the short presentation times. The accuracy of the face stimulus timings was verified using a photodiode and oscilloscope. Faces were selected from the NimStim Set of Facial Expressions [35].

### fMRI Data Acquisition

Functional and structural MRI scans were acquired on a General Electric 3.0 Tesla scanner (GE Signa, Milwaukee, WI) with an 8-channel phased-array head coil. The fMRI was performed using a gradient-echo echoplanar imaging (EPI) pulse sequence (39 continuous slices, TE = 20 ms, TR = 2000 ms, flip angle = 90°, 64×64 matrix, field of view = 22 cm, voxel dimensions = 3.4×3.4×3.0 mm^3^). A total of 290 fMRI images were obtained during each of four 10-minute runs while participants performed the task. Four images were discarded at the beginning of each run to allow for steady-state tissue magnetization. A high-resolution anatomical scan was obtained during the same scan session using a fast spoiled gradient echo (FSPGR) sequence (TR = 780 ms, TE = 2.7 ms, flip angle = 12 degrees, FOV = 22 cm, matrix = 224×224, number of axial slices = 128, slice thickness = 1.2 mm, in-plane resolution = 0.98 mm^2^) to provide an anatomical framework for the functional imaging analyses.

### Analysis of Behavioral Performance

Behavioral performance data were analyzed using SPSS 14.0 (SPSS Inc., Chicago, IL). The accuracy of the subject's response to each stimulus presentation and reaction times for identification of face stimuli as a “target" face were recorded using E-Prime software. The validity and effectiveness of the backward masking task design were assessed and established as described in our initial report [8].

### Analysis of Functional Neuroimaging Data

Image analyses were performed using the general linear model within SPM5 (Wellcome Trust Center for Neuroimaging, London, England, http://www.fil.ion.ucl.ac.uk/spm). Whole brain fMRI volumes were realigned to the first volume, co-registered to each subject's anatomical MRI scan, normalized to the Montreal Neurological Institute (MNI) standard brain template and smoothed using an 8 mm full-width at half-maximum Gaussian kernel. A high-pass with a cutoff period of 128 sec was used to correct low-frequency artifacts, serial correlations were corrected by choosing an autoregressive model of the order 1 [AR(1)] model, and the non-specific effects of global fluctuations in blood-oxygen-level dependent (BOLD) signal were removed using global normalization. The realignment parameters were modeled into the analysis as regressors of no interest to correct the image data for motion artifacts. Data for a task run were removed from the analyses if the subject showed movement of more than one-half voxel (1.5 mm) translation or 1.25° rotation. The fMRI data from three or four runs were included for all participants.

Single-subject t-contrast maps within the groups (HC, MDD) were generated by computing the difference maps between each emotional condition (SN, HN, NN) versus baseline (crosshair image). For the group level analysis, a 2×3 (group×emotion) ANOVA was performed using a random-effects analysis of the eigenvariate values generated from the single-subject analyses to assess the hemodynamic differences across conditions. *Post hoc* t-tests were conducted to further evaluate differences between the MDD and HC groups by comparing specific contrasts involving each of the emotional face conditions (SN, HN, NN) versus baseline. A comparison of masked-sad versus masked-happy faces (SN-HN) was defined as (SN_MDD_ – HN_MDD_) versus (SN_HC_ –HN_HC_), a comparison of masked-sad versus masked-neutral faces (SN-NN) was defined as (SN_MDD_ –NN_MDD_) versus (SN_HC_ –NN_HC_), and finally a comparison of masked-happy versus masked-neutral faces (HN-NN) was defined as (HN_MDD_ –NN_MDD_) versus (HN_HC_ –NN_HC_). The significance threshold for a reported group difference in the mean regional BOLD signal was set at a height threshold for a cluster's peak voxel at p≤0.001 and a minimum cluster size of k = 23 voxels. The minimum cluster size was determined using Monte Carlo simulations (10,000 iterations) implemented in AFNI's AlphaSim program [36] to calculate the spatial extent threshold that would be equivalent to correcting for multiple comparison testing while maintaining a cluster-level false-positive detection rate of p<0.05. Regions that remained significant after applying this correction for multiple comparisons were indicated in the tables using an asterisk. In addition, results were reported in the tables that remained significant after using the same minimum cluster size applied in our earlier amygdala region-of-interest analysis, in order to afford continuity with our previously published results (i.e. 10 voxels-[8]). The coordinates for the voxel with the peak p-value within each cluster were transformed from MNI coordinates to the stereotaxic array of Talairach and Tournoux [37]. Anatomical localization was performed with reference to the Talairach and Tournoux [37] and Mai et al. [38] atlases.

## Results

The demographic and clinical characteristics of the study samples appear in Table 1. Subject groups were not significantly different in gender composition, mean age or mean intelligence quotients. Thirteen of the unmedicated MDD subjects were naïve to psychotropic drug treatment. For those subjects who previously had received antidepressant drug treatment the mean (SD) drug-free period was 21 (23) months (range 1 to 72 months). The mean (SD) age at illness onset was 16.7 (6.0) years and the mean (SD) number of major depressive episodes were 3.2 (2.0).

**Table 1:** Mean and standard deviation results for subject demographic characteristics and clinical symptom rating scales.

Group	n	Females	Age	WASI	HAM-D	ATQ	IDS-SR	STAI-S	STAI-T	TCQ-D	TCQ-W	TCQ-P
HC	25	60%	29 (6.7)	122 (9.3)	0.0 (0.2)	2.5 (3.0)	3.2 (3.3)	27 (7.1)	27 (6.4)	15 (3.1)	7.6 (1.5)	6.9 (1.1)
MDD	22	55%	31 (7.8)	120 (14)	24^a^ (6.3)	65^a^ (24)	32^a^ (7.2)	51^a^ (8.3)	61^a^ (6.2)	12^a^ (1.9)	11^a^ (2.6)	10^a^ (2.9)

a MDD>HC, p<0.001.

Abbreviations: HC = healthy control; MDD = current major depressive disorder; WASI = Weschler Abbreviated Scale of Intelligence; HAM-D = Hamilton Depression Rating Scale; ATQ = Automatic Thoughts Questionnaire; IDS-SR = Inventory of Depressive Symptoms- Self-Rating; STAI-S = State-Trait Anxiety Inventory- State; STAI-T = State-Trait Anxiety Inventory- Trait; TCQ-D = Thought Control Questionnaire- distraction subscale; TCQ-W = Thought Control Questionnaire- worry subscale; TCQ-P = Thought Control Questionnaire- punishment subscale.

### Behavioral Performance on the fMRI task

Upon debriefing after the scanning session, participants reported no awareness of the backward masking technique (i.e., they denied perception of the masked face stimuli). Participants performed at chance level for the detection of target faces that had been presented in the masked face position [8].

The behavioral manifestation of the negative affective bias was demonstrated using a group×masked-condition interaction in the reaction time data, previously described in [8]. The reaction time for target faces was faster to masked-happy faces versus masked-sad faces in controls (p<0.001) and was faster to masked-sad faces versus masked-happy faces in currently-depressed subjects versus controls (p<0.05).

### fMRI Results

**Figure 1:**
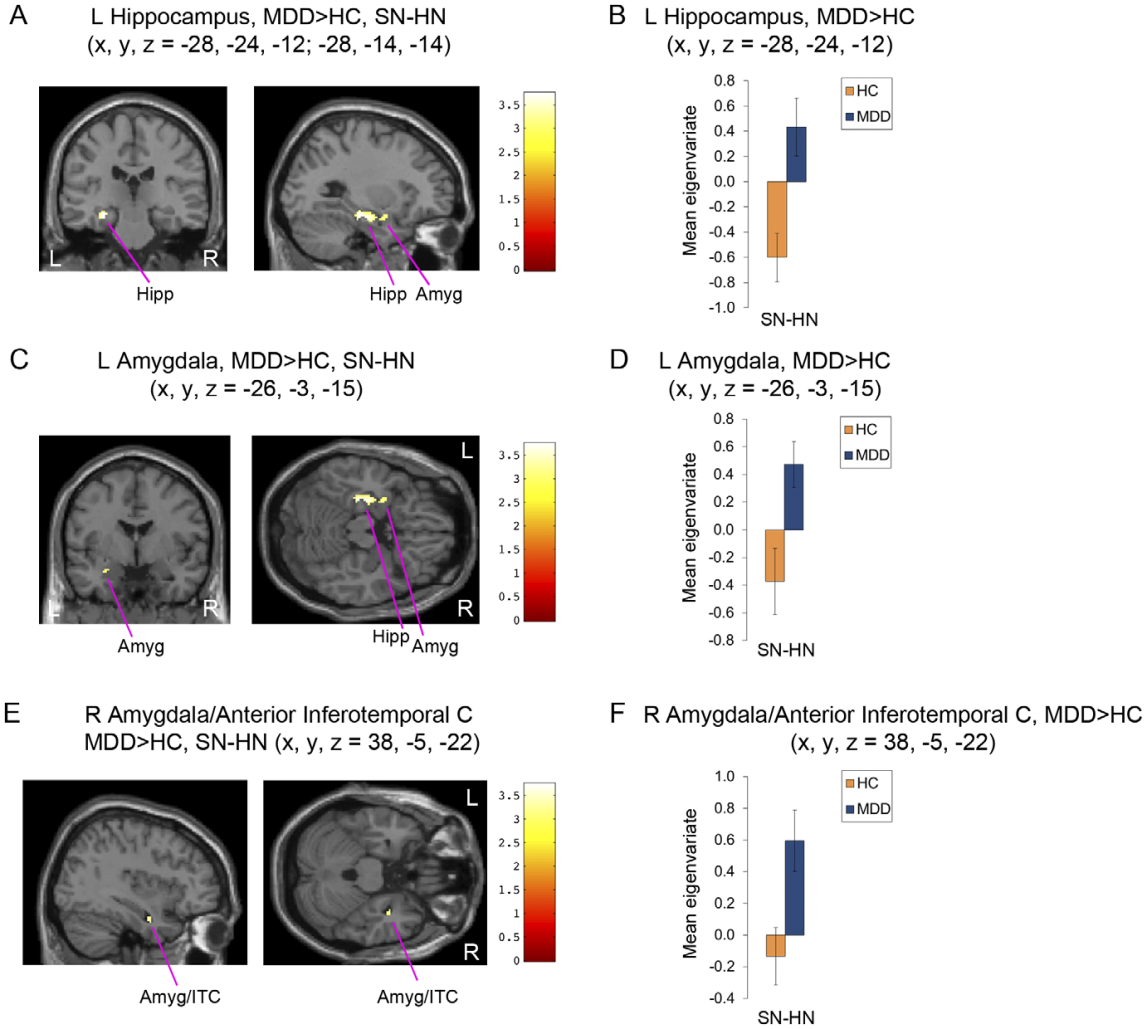
Neuroimaging results for masked-sad versus masked-happy faces (SN-HN), MDD>HC. (a–f) Statistical parametric map sections and corresponding bar graphs including standard error bars of contrast eigenvariates showing differences in the hemodynamic response to SN-HN between MDD and HC subjects for loci identified within the ANOVA: (a,b) left hippocampus, (c,d) left amygdala, (e,f) right amygdala/anterior inferotemporal cortex. Coordinates for the peak T-values for differences in the response to SN-HN correspond to the stereotaxic array of Talairach and Tournoux [37] as the distance in mm from the anterior commissure (positive x = right, positive y = anterior, positive z = dorsal). Abbreviations: L = left; R = right; MDD = major depressive disorder; HC = healthy control; C = cortex; SN = masked-sad faces; HN = masked-happy faces; SN-HN = masked-sad versus masked-happy faces; Hipp = hippocampus; Amyg = amygdala; ITC = inferotemporal cortex.

**Figure 2:**
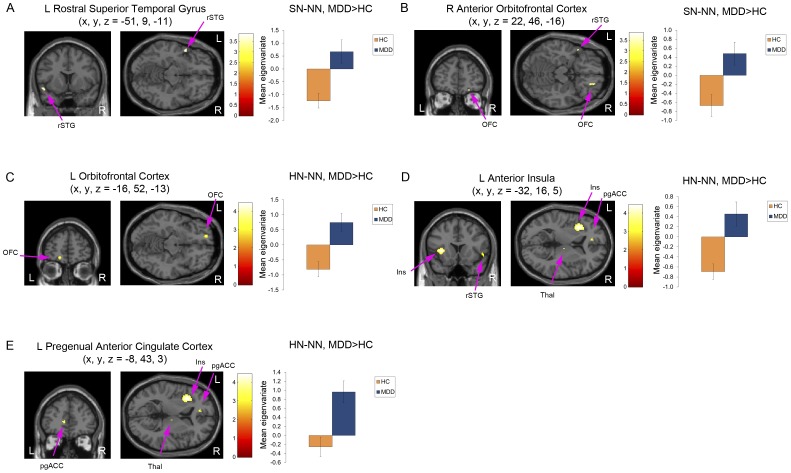
Neuroimaging results for masked-sad versus masked-neutral faces (SN-NN) and masked-happy versus masked-neutral faces (HN-NN). (a–b) Voxels showing differences in the hemodynamic response to SN-NN between MDD and HC subjects in the (a) left rostral superior temporal gyrus and (b) right anterior orbitofrontal cortex. (c–e) Voxels showing differences in the hemodynamic response to HN-NN between MDD and HC subjects in the (c) left orbitofrontal cortex, (d) left anterior insula, and (e) left pregenual anterior cingulate cortex. Bar graphs of the contrast eigenvariates with standard error bars are shown to the right of the corresponding statistical parametric map images in MDD versus HC subjects for the loci identified within the ANOVA (a–e). Abbreviations: L = left; R = right; MDD = major depressive disorder; HC = healthy control, SN = masked-sad faces; HN = masked-happy faces; NN = masked-neutral faces; SN-NN = masked-sad versus masked-neutral faces; HN-NN = masked-happy versus masked-neutral faces; rSTG = rostral superior temporal gyrus; OFC = orbitofrontal cortex; Ins = insula; latOFC = lateral orbitofrontal cortex; Thal = thalamus; pgACC = pregenual anterior cingulate cortex.

Analysis of the neuroimaging data indicated a significant group (MDD, HC)×emotion (SN, HN, NN) interaction in the left anterior insula (F_2,90_ = 9.63, p<0.001; x = −32, y = 16, z = 5), left rostral superior temporal gyrus (rSTG; F_2,90_ = 7.62, p = 0.001; x = −51, y = 9, z = −11) and left hippocampus (F_2,90_ = 7.34, p = 0.001; x = −28, y = −24, z = −12). Tables 2 and 3 list results of the t-tests performed to characterize the specific contrasts that accounted for the significant interactions. Within the group×emotion model these tests evaluated the significance of differences between masked-sad versus masked-happy faces (SN-HN), masked-sad versus masked-neutral faces (SN-NN) and masked-happy versus masked-neutral faces (HN-NN). The eigenvariate values were extracted from each significant cluster's peak and reported in Figures 1, 2, and Tables S1, S2, S3.

**Table 2:** Regional differences in the BOLD response to masked-sad faces versus masked-happy faces (SN-HN) in patients with major depressive disorder and healthy controls.

Condition	Region	SN-HN				
		Stereotaxic Coordinates			Cluster size*	Z-value#
		X	Y	Z		
MDD>HC	L Hippocampus	−28	−24	−12	146*	3.60
	L Hippocampus	−28	−14	−14		3.47
	L Amygdala	−26	−3	−15		3.10∧
	R Amygdala/Anterior Inferotemporal C	38	−5	−22	12	3.18

Abbreviations: L = left; R = right; MDD = major depressive disorder; HC = healthy control; SN = masked-sad faces; HN = masked-happy faces; SN-HN = masked-sad versus masked-happy faces; C = cortex.

* The cluster size was significant after applying a cluster threshold of k = 23 voxels using Monte Carlo simulations implemented in AFNI's AlphaSim program to correct for multiple comparisons.

# The statistical t-values for these regions are reported in the text, while corresponding z-values are reported in the table.

∧ The peak amygdala coordinate varies slightly from those reported in our previous paper [8] because here we report results for SN-HN based on a different statistical approach (2×3 ANOVA).

**Table 3:** Regional differences in the BOLD response to masked-sad faces versus masked-neutral faces (SN-NN) and masked-happy versus masked-neutral faces (HN-NN) in patients with major depressive disorder and healthy controls.

Condition	Region	SN-NN					HN-NN				
		Stereotaxic coordinates			Cluster size*	Z-value	Stereotaxic coordinates			Cluster size*	Z-value#
		X	Y	Z			X	Y	Z		
MDD>HC	L Rostral STG	−51	9	−11	24*	3.68	−53	4	2	19	3.19
	R Rostral STG	51	0	0	17	3.10	51	15	−2	22	3.18
	R Anterior OFC	22	46	−16	10	3.18					
	L Anterior OFC						−16	52	−13	22	3.37
	L Anterior Insula						−32	16	5	291*	4.20
	L Pregenual ACC						−8	43	3	13	3.24
	R Ventral Thalamus						10	−13	4	22	3.17
	R Postcentral G						65	−24	31	10	3.08
HC>MDD	L Inferior Parietal C	−44	−44	50	136*	3.40					
	L Inferior Parietal C	−49	−38	46		3.22					
	R Frontal Polar C	34	60	4	23*	3.32					
	L Middle Occipital G						−44	−81	15	14	3.32

Abbreviations: L = left; R = right; MDD = major depressive disorder; HC = healthy control; SN = masked-sad faces; HN = masked-happy faces; NN = masked-neutral faces; SN-NN = masked-sad versus masked-neutral faces; HN-NN = masked-happy versus masked-neutral faces; STG = superior temporal gyrus; OFC = orbitofrontal cortex; C = cortex; ACC = anterior cingulate cortex; G = gyrus; PFC = prefrontal cortex.

* The cluster size was significant after applying a cluster threshold of k = 23 voxels using Monte Carlo simulations implemented in AFNI's AlphaSim program to correct for multiple comparisons.

# The statistical t-values for these regions are reported in the text, while corresponding z-values are reported in the table.

For the SN-HN contrast, the between conditions difference in BOLD activity was greater for the depressed subjects versus the controls in the left hippocampus (t_90_ = 3.74, p<0.001; Figure 1a–b), left amygdala (t_90_ = 3.20, p = 0.001; Figure 1c–d) and right amygdala/anterior inferotemporal cortex (t_90_ = 3.28, p = 0.001; Figure 1e–f). No region was identified in which the BOLD response obtained under the SN-HN contrast was greater in the controls than in the depressed subjects.

For the SN-NN contrast, the between conditions difference in BOLD activity was greater for the depressed subjects versus the controls in left and right rSTG (t_90_ = 3.84, p<0.001 and t_90_ = 3.19, p = 0.001, respectively; Figure 2a) and right anterior orbitofrontal cortex (OFC; t_90_ = 3.28, p = 0.001; Figure 2b). Conversely, the mean BOLD activity was greater for the controls than the depressed subjects in the left inferior parietal cortex (t_90_ = 3.52, p<0.001) and right frontal polar cortex (t_90_ = 3.43, p<0.001).

Finally, for the HN-NN contrast, the between conditions difference in BOLD activity was greater in MDD versus HC in the left anterior OFC (t_90_ = 3.49, p<0.001; Figure 2c) left anterior insula (t_90_ = 4.42, p<0.001; Figure 2d), left pregenual anterior cingulate cortex (pgACC; t_90_ = 3.35, p = 0.001; Figure 2e), left and right rSTG (t_90_ = 3.29, p = 0.001 and t_90_ = 3.28, p = 0.001, respectively), right ventral thalamus (t_90_ = 3.27, p = 0.001), and right postcentral gyrus (t_90_ = 3.18, p = 0.001). The between conditions difference in BOLD activity was greater in the controls than in the depressives in the HN-NN contrast in the left middle occipital gyrus (t_90_ = 3.43, p<0.001).

Exploratory analyses were performed *post hoc* to characterize potential relationships between the neuroimaging findings and the clinical assessments of illness course or severity in the MDD subjects. Spearman rank order correlation coefficients were computed to explore associations between the eigenvariate values and the total number of major depressive episodes, the age at illness onset, and the HAM-D, IDS-SR, ATQ, TCQ and STAI scores. The HAM-D scores correlated inversely with the hemodynamic response to HN-NN in the right rSTG and left anterior insula, such that the response to masked-happy faces decreased as depression severity increased (r = −0.43, p<0.05 and r = −0.51, p<0.05, respectively; Figures S1a–S1b). In contrast, the IDS-SR, ATQ and STAI-T scale scores positively correlated with the hemodynamic response to SN-NN in the right rSTG, such that the response to masked-sad faces increased as the self-reported depression severity (IDS-SR), frequency of automatic negative thoughts (ATQ) and trait levels of anxiety (STAI-T) increased (r = 0.66, p<0.001; r = 0.55, p<0.01 and r = 0.49, p<0.05, respectively; Figures S1c–S1e). In addition, for the HN-NN condition, the age at onset correlated inversely with the hemodynamic response to masked-happy faces in the left anterior insula (r = −0.45, p<0.05) and the STAI-State scores correlated positively with the hemodynamic response in the left anterior orbitofrontal cortex (r = 0.49, p<0.05; Figures S1f–S1g).

## Discussion

These data identify neuroanatomical regions that, in addition to the amygdala, show changes in hemodynamic activity associated with automatic negative emotional processing biases in unmedicated, currently-depressed subjects with MDD. During exposure to masked-sad versus masked-happy faces, the depressed group showed greater BOLD activity than the control group in the left hippocampus and right anterior inferotemporal cortex, in addition to the left amygdala (Table 2). During exposure to masked-sad versus masked-neutral faces, the depressed group showed greater BOLD activity than the control group in the bilateral rSTG and right anterior OFC (Table 3). In the hippocampus, amygdala, rSTG and right anterior OFC, the group difference was attributable *both* to the depressed group showing a *higher* BOLD response to masked-sad versus masked-happy or neutral faces, *and* to the control group showing a *lower* BOLD response to masked-sad versus masked-happy or neutral faces (Figures 1 and 2, Tables S1, S2). Finally, the BOLD response was higher in the depressed subjects but lower in the controls to masked-happy versus masked-neutral faces in the bilateral rSTG, *left* anterior OFC, left anterior insula, left pregenual ACC, right ventral thalamus and right postcentral gyrus (Figure 2, Table S3). These data thus revealed several structures in which the hemodynamic response patterns appeared opposite in direction between groups for masked-sad or masked-happy faces relative to masked-neutral faces, implying that a shared neural circuitry may be processing stimuli distinctly between depressives and controls depending upon the emotional valence of the stimuli.

Several of the regions implicated in this study reflect structures that share substantial monosynaptic projections with the amygdala. The networks formed by these projections include the anterior inferotemporal cortex that participates in visual processing [39]–[43], and the networks formed between the medial and orbital prefrontal cortex, amygdala, hippocampus, and rSTG [21], [22], [43], [44] that play roles in integrating sensory information and modulating the behavioral, emotional and visceral responses to salient stimuli [21], [45], [46]. The most striking hemodynamic changes with respect to their effect size and the direction of the changes paralleling those within the amygdala included the hippocampus, right anterior OFC, and bilateral rSTG. Each of these structures manifested an exaggerated BOLD response to masked-sad faces relative to masked-happy or masked-neutral faces in the depressed subjects. The projections from these regions putatively convey information to the amygdala regarding context (hippocampus) and sensory integration (anterior OFC, parts of rSTG) [21], [47]–[49]. In contrast, the regions that showed hemodynamic responses that were opposite in direction to those seen in the amygdala, namely the left pregenual anterior cingulate cortex, left anterior OFC (BA 10o) and bilateral areas of the rSTG form part of the extended medial prefrontal, or “visceromotor", network [21], [49]. The monosynaptic projections from several regions within the visceromotor network have been characterized for their role in influencing the outflow of amygdala activity related to emotional expression and to tuning cortical neuronal responses to sensory stimuli [50].

Most previous neuroimaging studies investigating the functional anatomical correlates of emotional processing biases have focused on the amygdala. This structure contains cells that are selectively tuned to stimulus characteristics that allow for the early detection of salient information, including faces [18], [51]. Moreover, the amygdala projections to the primary sensory cortices both directly and indirectly through the cholinergic inputs originating in the basal forebrain tune the firing parameters of neurons in the sensory cortices so they become differentially sensitive to salient sensory signals [52], [53]. These interactions between the sensory cortices and the lateral nucleus of the amygdala also are modulated by input from hippocampal and orbitofrontal cortical projections during the associative learning processes that underlie appetitive and aversive conditioning [54], [55]. The extended network formed by projections between these structures thus integrates limbic and sensory input as part of assessing stimulus valence and incentive value within a particular context, based upon the tendency for a stimulus to be associated with rewarding or punishing reinforcement [21], [22], [47], [55]. The differential hemodynamic responses of the hippocampus and OFC to implicitly presented sad and happy face stimuli observed between depressives and controls thus may reflect and/or mediate the differences in stimulus salience that maintain a positive processing bias in healthy subjects, but a negative bias in depressed subjects. Although the causal mechanism underlying the distinct directions in the emotional bias between groups remains unknown, the circuits involving these structures appear to be responding automatically to the salience of the happy face in healthy individuals, but responding automatically to the salience of the sad face in depression.

In the current study, the group differences that were greatest with respect to statistical significance localized to the hippocampus and anterior insula (Figures 1a, 2e). Lesions of the hippocampus interrupt afferent neurotransmission to the amygdala that conveys information regarding environmental contexts that hold emotional salience [47], [56]. Thus it is conceivable that the hippocampus plays a role in MDD in *setting the context for a negative emotional processing bias*. In other words the hippocampal input may specifically drive the amygdala to respond differently to sad versus happy stimuli within the context of the depressive episode.

Notably, the anterior insula changed activity most robustly in response to masked-happy faces (HN versus NN), a condition under which the BOLD signal increased in the depressives but decreased in the controls. This pattern of group differences thus was opposite to that observed in the amygdala. In healthy humans the anterior insula consistently activates in response to stimuli that are aversive or disgusting, or to face stimuli that show expressions of disgust [57], [58]. These data, taken together with our finding that depressed subjects show increased BOLD activity in this region in response to happy faces, suggest the hypothesis that pathological activation of the anterior insula plays a role in diverting attention away from positive stimuli in MDD.

In healthy volunteers previous research had shown greater BOLD responses in the amygdala [16], [17] and the ACC [16] to masked-happy faces relative to either fixation point or to masked-sad or neutral face control conditions. In our original report comparing HC to MDD participants [8], we replicated these findings in the amygdala in the healthy subjects, who showed a greater BOLD response to masked-happy faces versus masked-sad faces. Here we report a pregenual ACC area (located>one cm from the ACC areas described in Killgore et al. [17] that showed a greater response to masked-happy versus neutral faces in depressed subjects versus healthy controls (Figure 2e).

A previous study by Suslow et al. [9] in antidepressant-medicated but persistently depressed MDD subjects also showed hemodynamic responses in the *right* amygdala that were exaggerated to masked-sad faces and blunted to masked-happy faces, compatible with our findings in *unmedicated* depressed subjects in this region. Suslow et al. [9] additionally reported the results of a whole brain analysis in which they observed significant group-by-emotion interaction effects in the left anterior insula and left STG, structures where we also observed significant group-by-emotion interactions. Although Suslow et al. [9] did not further characterize these interactions, we found here that the interaction in left anterior insula was attributable to a greater BOLD response to masked-happy versus masked-neutral faces in depressives compared to controls, while the interaction in left STG was accounted for by a greater response to masked-sad versus neutral faces in depressives compared to controls.

The manifestation of MDD may result partly as a consequence of a pathological change in the top-down control from the medial prefrontal cortical network over structures in the limbic system, such as the amygdala and hippocampus (see [24]). Impairment in top-down modulatory control exerted by the medial network over amygdala neuronal activity conceivably may result in exaggerated processing of aversive or potentially threatening emotional stimuli, together with attenuated processing of potentially positive or rewarding stimuli [49], [59].

The cross-sectional design of this study did not allow us to address directly whether the findings presented herein are mood state-dependent, or instead reflect trait-like abnormalities that precede and/or persist across mood episodes. Nevertheless, our work has shown an increased hemodynamic response to masked-sad faces vs. masked-happy faces in the amygdala [8] and hippocampus (unpublished data) in unmedicated-remitted subjects with MDD, suggesting that the emotional processing bias manifest in these structures may constitute a persistent bias toward the processing of negative stimuli. Further evidence of a trait-like bias in the processing of negative information has been reported for subjects with genetic risk carrying risk alleles in the 5-HTTLPR polymorphism (see [60]), including for non-consciously presented stimuli [61], and in healthy subjects with environment risk suffering from childhood maltreatment [62]. Additionally, however, there is strong evidence for the hypothesis [63] that antidepressant drugs exert their effects by normalizing the bias toward negative information processing (e.g. [8], [12], [15]). While this normalization may appear to provide support for a processing bias associated with the current mood state, additional work is needed to assess whether or not this bias represents a biomarker underlying the vulnerability to relapse in MDD that can be modulated by antidepressant drug treatment. A longitudinal study of individuals at high familial risk for MDD may assist in determining the developmental time course of these emotional processing biases associated with MDD.

Other limitations of our study design also merit comment. First, there is no explicitly *modeled* baseline in the slow event-related design of the task. The stimulus contrasts in the analyses compared one emotional stimulus type directly to another stimulus type that differed only in their emotional valence. The slow event-related design was selected to allow the hemodynamic response to return to baseline and to reduce overlap of the neural response to each stimulus condition. However, the result is additional noise in the baseline measure estimates, thereby making reductions relative to baseline more difficult to interpret (Supplementary Tables S1, S2, S3). Nevertheless, the results highlight the robustness of the difference in emotional conditions compared to baseline. A rapid event-related design conceivably may allow for better modeling of a specific baseline measure within the design construct because more trials can be completed within the scanning session. Second, the study design did not allow determination of a causal relationship between the abnormal BOLD responses identified herein and the pathogenesis of MDD. Finally, this study did not address the generalizability of the results to other categories of mood disorders and did not assess potential sex differences in the processing of emotional information in MDD.

In conclusion, our findings provide support for automatic neurophysiological biases toward negative and away from positive emotional stimuli in MDD, and show that these processing biases are supported by an extended network of structures known to share substantial projections with the amygdala and to participate in the evaluation of stimulus salience. Future studies are needed to explore whether the abnormal hemodynamic response pattern to negative versus positive stimuli in MDD can sensitively and specifically discriminate individuals with mood disorders from healthy controls, and to characterize the potential relationship of this pattern with the onset, maintenance and recurrence of depressive episodes.

## Supporting Information

Figure S1 Relationships between the neuroimaging findings and the clinical assessments of illness course or severity in MDD participants: (a,b) Hamilton Depression Rating Scale, (c) Inventory of Depressive Symptomatology, (d) Automatic Thoughts Questionnaire, (e) State-Trait Anxiety Inventory- Trait Score, (f) age of onset and (g) State-Trait Anxiety Inventory- State Score.
(TIF)

Table S1 Mean (SD) eigenvariates extracted from the peak voxel regions where hemodynamic activity was significantly different between healthy controls and participants with major depressive disorder for masked-sad faces (SN) versus masked-happy faces (HN). The coordinates correspond to regions reported in Table 2 of the manuscript.
(DOCX)

Table S2 Mean (SD) eigenvariates extracted from the peak voxel regions where hemodynamic activity was significantly different between healthy controls and participants with major depressive disorder for masked-sad faces (SN) versus masked-neutral faces (NN). The coordinates correspond to regions reported in Table 3 of the manuscript.
(DOCX)

Table S3 Mean (SD) eigenvariates extracted from the peak voxel regions where hemodynamic activity was significantly different between healthy controls and participants with major depressive disorder for masked-happy faces (HN) versus masked-neutral faces (NN). The coordinates correspond to regions reported in Table 3 of the manuscript.
(DOCX)

## References

[1] Mogg K, Bradbury KE, Bradley BP (2006) Interpretation of ambiguous information in clinical depression. Beh Res Ther 44: 1411–1419. 10.1016/j.brat.2005.10.008 16487479

[2] Gotlib IH, Kasch KL, Traill S, Joorman J, Arnow BA, et al (2004) Coherence and specificity of information-processing biases in depression and social phobia. J Abnorm Psychol 113: 386–398. 15311984 10.1037/0021-843X.113.3.386

[3] Gotlib IH, Krasnoperova E, Yue DN, Joormann J (2004) Attentional biases for negative interpersonal stimuli in clinical depression. J Abnorm Psychol 113: 121–135. 14992665 10.1037/0021-843X.113.1.121

[4] Elliott R, Rubinsztein JS, Sahakian BJ, Dolan RJ (2000) Selective attention to emotional stimuli in a verbal go/no-go task: an fMRI study. Neuroreport 11: 1739–1744. 10852235 10.1097/00001756-200006050-00028

[5] Murray LA, Whitehouse WG, Alloy LB (1999) Mood congruence and depressive deficits in memory: a forced-recall analysis. Memory 7: 175–196. 10645378 10.1080/741944068

[6] Murphy FC, Sahakian BJ, Rubinsztein JS, Michael A, Rogers RD, et al (1999) Emotional bias and inhibitory control processes in mania and depression. Psychol Med 29: 1307–1321. 10616937 10.1017/s0033291799001233

[7] Bradley BP, Mogg K, Williams R (1995) Implicit and explicit memory for emotion-congruent information in clinical depression and anxiety. Beh Res Ther 33: 755–770. 10.1016/0005-7967(95)00029-w 7677713

[8] Victor TA, Furey ML, Fromm SJ, Öhman A, Drevets WC (2010) Relationship between amygdala responses to masked faces and mood state and treatment in major depressive disorder. Arch Gen Psychiatry 67: 1128–1138. 21041614 10.1001/archgenpsychiatry.2010.144 PMC3253452

[9] Suslow T, Konrad C, Kugel H, Rumstadt D, Zwitserlood P, et al (2010) Automatic mood-congruent amygdala responses to masked facial expressions in major depression. Biol Psychiatry 67: 155–160. 19748075 10.1016/j.biopsych.2009.07.023

[10] Siegle GJ, Thompson W, Carter CS, Steinhauer SR, Thase ME (2007) Increased amygdala and decreased dorsolateral prefrontal BOLD responses in unipolar depression: related and independent features. Biol Psychiatry 61: 198–209. 17027931 10.1016/j.biopsych.2006.05.048

[11] Surguladze S, Brammer MJ, Keedwell P, Giampietro V, Young AW, et al (2005) A differential pattern of neural response toward sad versus happy facial expressions in major depressive disorder. Biol Psychiatry 57: 201–209. 15691520 10.1016/j.biopsych.2004.10.028

[12] Fu CH, Williams SC, Cleare AJ, Brammer MJ, Walsh ND, et al (2004) Attenuation of the neural response to sad faces in major depression by antidepressant treatment: a prospective, event-related functional magnetic resonance imaging study. Arch Gen Psychiatry 61: 877–889. 15351766 10.1001/archpsyc.61.9.877

[13] Siegle GJ, Steinhauer SR, Thase ME, Stenger VA, Carter CS (2002) Can't shake that feeling: event-related fMRI assessment of sustained amygdala activity in response to emotional information in depressed individuals. Biol Psychiatry 51: 693–707. 11983183 10.1016/s0006-3223(02)01314-8

[14] Drevets WC, Gautier C, Lowry T, Bogers W, Greer P, et al (2001) Abnormal hemodynamic responses to facially expressed emotion in major depression. Soc Neurosci Abstr 27: 785.1.

[15] Sheline YI, Barch DM, Donnelly JM, Ollinger JM, Snyder AZ, et al (2001) Increased amygdala response to masked emotional faces in depressed subjects resolves with antidepressant treatment: an fMRI study. Biol Psychiatry 50: 651–658. 11704071 10.1016/s0006-3223(01)01263-x

[16] Juruena MF, Giampietro VP, Smith SD, Surguladze SA, Dalton JA, et al (2010) Amygdala activation to masked happy facial expressions. J Int Neuropsychol Soc 16: 383–387. 19958569 10.1017/S1355617709991172

[17] Killgore WD, Yurgelun-Todd DA (2004) Activation of the amygdala and anterior cingulate during nonconscious processing of sad versus happy faces. NeuroImage 21: 1215–1223. 15050549 10.1016/j.neuroimage.2003.12.033

[18] Morris JS, Öhman A, Dolan RJ (1999) A subcortical pathway to the right amygdala mediating “unseen" fear. Proc Natl Acad Sci U S A 96: 1680–1685. 9990084 10.1073/pnas.96.4.1680 PMC15559

[19] LeDoux JE (1996) The Emotional Brain: The Mysterious Underpinnings of Emotional Life: Simon and Schuster. 384 p.

[20] Liddell BJ, Brown KJ, Kemp AH, Barton MJ, Das P, et al (2005) A direct brainstem-amygdala-cortical ‘alarm’ system for subliminal signals of fear. NeuroImage 24: 235–243. 15588615 10.1016/j.neuroimage.2004.08.016

[21] Ongur D, Ferry AT, Price JL (2003) Architectonic subdivision of the human orbital and medial prefrontal cortex. J Comp Neurol 460: 425–449. 12692859 10.1002/cne.10609

[22] Ongur D, Price JL (2000) The organization of networks within the orbital and medial prefrontal cortex of rats, monkeys and humans. Cereb Cortex 10: 206–219. 10731217 10.1093/cercor/10.3.206

[23] Drevets WC, Price JL, Furey ML (2008) Brain structural and functional abnormalities in mood disorders: implications for neurocircuitry models of depression. Brain Struct Funct 213: 93–118. 18704495 10.1007/s00429-008-0189-x PMC2522333

[24] Savitz J, Drevets WC (2009) Bipolar and major depressive disorder: neuroimaging the developmental-degenerative divide. Neurosci and Biobehav Rev 33: 699–771. 19428491 10.1016/j.neubiorev.2009.01.004 PMC2858318

[25] American Psychiatric Association (1994) Diagnostic and Statistical Manual of Mental Disorders (DSM-IV): American Psychiatric Press.

[26] Oldfield RC (1971) The assessment and analysis of handedness: the Edinburgh inventory. Neuropsychologia 9: 97–113. 5146491 10.1016/0028-3932(71)90067-4

[27] First MB, Spitzer RL, Gibbon M, Williams JBW (2002) Structured Clinical Interview for DSM-IV-TR Axis I Disorders, Research Version, Patient Edition (SCID-I/P): New York State Psychiatric Institute, Biometrics Research.

[28] Maxwell E (1992) Family Interview for Genetic Studies (FIGS): A Manual for FIGS: Clinical Neurogenetics Branch, Intramural Research Program, National Institute of Mental Health.

[29] Wechsler D (1999) Wechsler Abbreviated Scale of Intelligence (WASI): Harcourt Assessment.

[30] Hamilton M (1960) A rating scale for depression. J Neurol Neurosurg Psychiatry 23: 56–62. 14399272 10.1136/jnnp.23.1.56 PMC495331

[31] Hollon SD, Kendall PC (1980) Cognitive self-statements in depression: development of an automatic thoughts questionnaire. Cognit Ther Res 4: 383–395.

[32] Rush AJ, Gullion CM, Basco MR, Jarrett RB, Trivedi MH (1996) The Inventory of Depressive Symptomatology (IDS): psychometric properties. Psychol Med 26: 477–486. 8733206 10.1017/s0033291700035558

[33] Spielberger CD, Gorsuch RL, Lushene RE (1970) Manual for the State-Trait Anxiety Inventory: Consulting Psychologists Press.

[34] Wells A, Davies MI (1994) The Thought Control Questionnaire: a measure of individual differences in the control of unwanted thoughts. Beh Res Ther 32: 871–878. 10.1016/0005-7967(94)90168-6 7993332

[35] Tottenham N, Tanaka JW, Leon AC, McCarry T, Nurse M, et al (2009) The NimStim set of facial expressions: judgments from untrained research participants. Psychiatry Res 168: 242–249. 19564050 10.1016/j.psychres.2008.05.006 PMC3474329

[36] Forman SD, Cohen JD, Fitzgerald M, Eddy WF, Mintun MA, et al (1995) Improved assessment of significant activation in functional magnetic resonance imaging (fMRI): use of a cluster-size threshold. Magn Reson Med 33: 636–647. 7596267 10.1002/mrm.1910330508

[37] Talairach J, Tournoux P (1988) Co-planar stereotaxic atlas of the human brain: 3-dimensional proportional system: an approach to cerebral imaging. Stuttgart, New York: Thieme Medical Publishers, Inc. 122 p.

[38] Mai JK, Assheuer J, Paxinos G (2003) Atlas of the Human Brain. London: Elsevier Academic Press. 246 p.

[39] Amaral DG, Price JL (1984) Amygdalo-cortical projections in the monkey (Macaca fascicularis). J Comp Neurol 230: 465–496. 6520247 10.1002/cne.902300402

[40] Amaral DG, Insausti R (1992) Retrograde transport of D-[3H]-aspartate injected into the monkey amygdaloid complex. Exp Brain Res 88: 375–388. 1374347 10.1007/BF02259113

[41] Amaral DG, Behniea H, Kelly JL (2003) Topographic organization of projections from the amygdala to the visual cortex in the macaque monkey. Neuroscience 118: 1099–1120. 12732254 10.1016/s0306-4522(02)01001-1

[42] Freese JL, Amaral DG (2005) The organization of projections from the amygdala to visual cortical areas TE and V1 in the macaque monkey. J Comp Neurol 486: 295–317. 15846786 10.1002/cne.20520

[43] Carmichael ST, Price JL (1995) Limbic connections of the orbital and medial prefrontal cortex in macaque monkeys. J Comp Neurol 363: 615–641. 8847421 10.1002/cne.903630408

[44] Carmichael ST, Price JL (1996) Connectional networks within the orbital and medial prefrontal cortex of macaque monkeys. J Comp Neurol 371: 179–207. 8835726 10.1002/(SICI)1096-9861(19960722)371:2<179::AID-CNE1>3.0.CO;2-#

[45] Tekin S, Cummings JL (2002) Frontal-subcortical neuronal circuits and clinical neuropsychiatry: an update. J Psychosom Res 53: 647–654. 12169339 10.1016/s0022-3999(02)00428-2

[46] Drevets WC (2001) Neuroimaging and neuropathological studies of depression: implications for the cognitive-emotional features of mood disorders. Curr Opin Neurobiol 11: 240–249. 11301246 10.1016/s0959-4388(00)00203-8

[47] Phillips RG, LeDoux JE (1992) Differential contribution of amygdala and hippocampus to cued and contextual fear conditioning. Behav Neurosci 106: 274–285. 1590953 10.1037//0735-7044.106.2.274

[48] Phillips RG, LeDoux JE (1994) Lesions of the dorsal hippocampal formation interfere with background but not foreground contextual fear conditioning. Learn Mem 1: 34–44. 10467584

[49] Price JL, Drevets WC (2010) Neurocircuitry of mood disorders. Neuropsychopharmacology 35: 192–216. 19693001 10.1038/npp.2009.104 PMC3055427

[50] Quirk GJ, Likhtik E, Pelletier JG, Pare D (2003) Stimulation of medial prefrontal cortex decreases the responsiveness of central amygdala output neurons. J Neurosci 23: 8800–8807. 14507980 10.1523/JNEUROSCI.23-25-08800.2003 PMC6740415

[51] Bordi F, LeDoux J (1992) Sensory tuning beyond the sensory system: an initial analysis of auditory response properties of neurons in the lateral amygdaloid nucleus and overlying areas of the striatum. J Neurosci 12: 2493–2503. 1613543 10.1523/JNEUROSCI.12-07-02493.1992 PMC6575825

[52] Mesulam MM, Mufson EJ, Levey AI, Wainer BH (1983) Cholinergic innervation of cortex by the basal forebrain: cytochemistry and cortical connections of the septal area, diagonal band nuclei, nucleus basalis (substantia innominata), and hypothalamus in the rhesus monkey. J Comp Neurol 214: 170–197. 6841683 10.1002/cne.902140206

[53] Holland PC (2007) Disconnection of the amygdala central nucleus and the substantia innominata/nucleus basalis magnocellularis disrupts performance in a sustained attention task. Behav Neurosci 121: 80–89. 17324052 10.1037/0735-7044.121.1.80 PMC2241658

[54] Sarter M, Givens B, Bruno JP (2001) The cognitive neuroscience of sustained attention: where top-down meets bottom-up. Brain Res Rev 35: 146–160. 11336780 10.1016/s0165-0173(01)00044-3

[55] Kringelbach ML, Rolls ET (2004) The functional neuroanatomy of the human orbitofrontal cortex: evidence from neuroimaging and neuropsychology. Prog Neurobiol 72: 341–372. 15157726 10.1016/j.pneurobio.2004.03.006

[56] Peper M, Karcher S, Wohlfarth R, Reinshagen G, LeDoux JE (2001) Aversive learning in patients with unilateral lesions of the amygdala and hippocampus. Biol Psychology 58: 1–23. 10.1016/s0301-0511(01)00098-9 11473792

[57] Seubert J, Kellermann T, Loughead J, Boers F, Brensinger C, et al (2010) Processing of disgusted faces is facilitated by odor primes: a functional MRI study. NeuroImage 53: 746–756. 20627130 10.1016/j.neuroimage.2010.07.012

[58] Jabbi M, Bastiaansen J, Keysers C (2008) A common anterior insula representation of disgust observation, experience and imagination shows divergent functional connectivity pathways. PloS One 3: e2939. 18698355 10.1371/journal.pone.0002939 PMC2491556

[59] Davidson RJ (2002) Anxiety and affective style: role of prefrontal cortex and amygdala. Biol Psychiatry 51: 68–80. 11801232 10.1016/s0006-3223(01)01328-2

[60] Munafo MR, Brown SM, Hariri AR (2008) Serotonin transporter (5-HTTLPR) genotype and amygdala activation: a meta-analysis. Biol Psychiatry 63: 852–857. 17949693 10.1016/j.biopsych.2007.08.016 PMC2755289

[61] Dannlowski U, Ohrmann P, Bauer J, Deckert J, Hohoff C, et al (2008) 5-HTTLPR biases amygdala activity in response to masked facial expressions in major depression. Neuropsychopharmacology 33: 418–424. 17406646 10.1038/sj.npp.1301411

[62] Dannlowski U, Kugel H, Huber F, Stuhrmann A, Redlich R, et al (2012) Childhood maltreatment is associated with an automatic negative emotion processing bias in the amygdala. Hum Brain Mapp doi: 10.1002/hbm.22112 [Epub ahead of print]. 10.1002/hbm.22112 PMC6870128 22696400

[63] Harmer CJ (2008) Serotonin and emotional processing: does it help explain antidepressant drug action? Neuropharmacology 55: 1023–1028. 18634807 10.1016/j.neuropharm.2008.06.036

