# Supplementary figures and images for: The Extended Functional Neuroanatomy of Emotional Processing Biases for Masked Faces in Major Depressive Disorder
Source: PLoS One. 2012 Oct 8;7(10):e46439. doi: 10.1371/journal.pone.0046439 (PMC3466291; doi:10.1371/journal.pone.0046439)

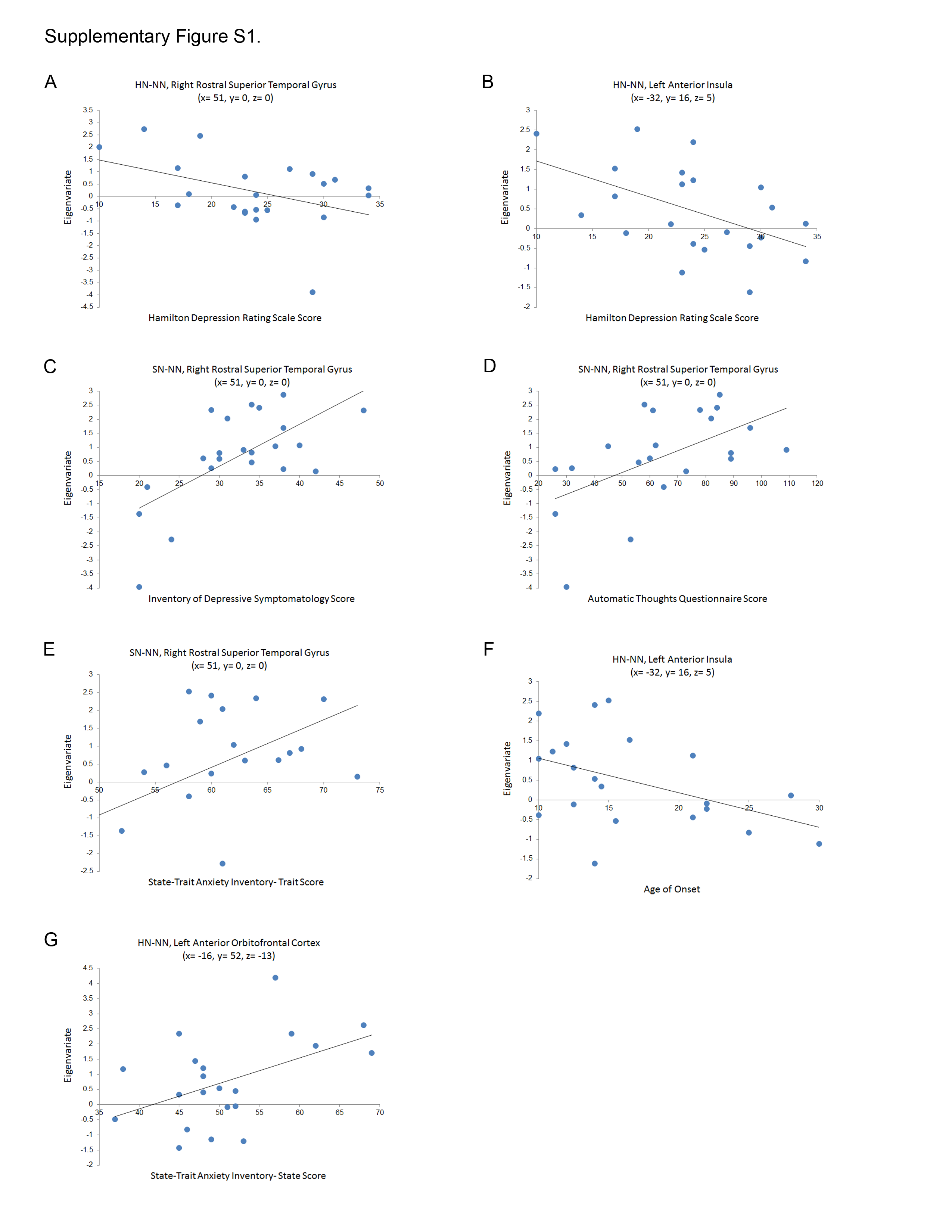

Supplement: Figure S1 — Relationships between the neuroimaging findings and the clinical assessments of illness course or severity in MDD participants: (a,b) Hamilton Depression Rating Scale, (c) Inventory of Depressive Symptomatology, (d) Automatic Thoughts Questionnaire, (e) State-Trait Anxiety Inventory- Trait Score, (f) age of onset and (g) State-Trait Anxiety Inventory- State Score. (TIF) [file pone.0046439.s001.tif]
