# Supplementary material for: The Extended Functional Neuroanatomy of Emotional Processing Biases for Masked Faces in Major Depressive Disorder
Source: PLoS One. 2012 Oct 8;7(10):e46439. doi: 10.1371/journal.pone.0046439 (PMC3466291; doi:10.1371/journal.pone.0046439)
Supplement: Table S1 — Mean (SD) eigenvariates extracted from the peak voxel regions where hemodynamic activity was significantly different between healthy controls and participants with major depressive disorder for masked-sad faces (SN) versus masked-happy faces (HN). The coordinates correspond to regions reported in Table 2 of the manuscript. (DOCX) [file pone.0046439.s002.docx]

Supplementary Table S1. Mean (SD) eigenvariates extracted from the peak voxel regions where hemodynamic activity was significantly different between healthy controls and participants with major depressive disorder for masked-sad faces (SN) versus masked-happy faces (HN). The coordinates correspond to regions reported in Table 2 of the manuscript.

| Region | x,y,z | Eigenvariate | | | | | |
| --- | --- | --- | --- | --- | --- | --- | --- |
|  |  | SN | | HN | | NN | |
|  |  | HC | MDD | HC | MDD | HC | MDD |
| MDD>HC, Masked-Sad Faces vs. Masked-Happy Faces | | | | | | | |
| L Hippocampus | -28, -24, -12 | -0.51 (0.55) | 0.10 (0.77) | 0.10 (0.60) | -0.34 (0.51) | -0.12 (0.52) | -0.19 (0.70) |
| L Hippocampus | -28, -14, -14 | -0.32 (0.57) | 0.02 (0.66) | 0.15 (0.61) | -0.43 (0.62) | -0.35 (0.50) | -0.47 (0.49) |
| L Amygdala | -26, -3, -15 | -0.17 (0.55) | 0.21 (0.74) | 0.20 (0.77) | -0.26 (0.41) | -0.03 (0.42) | -0.21 (0.49) |
| R Amygdala/  R Anterior ITC | 38, -5, -22 | -0.26 (0.42) | 0.08 (0.44) | -0.13 (0.64) | -0.52 (0.67) | -0.31 (0.55) | -0.40 (0.40) |

Abbreviations: SD= standard deviation; SN= masked-sad faces; HN= masked-happy faces; HC= healthy control; MDD= major depressive disorder; L= left; R= right; ITC= inferotemporal cortex
