# Supplementary material for: The Extended Functional Neuroanatomy of Emotional Processing Biases for Masked Faces in Major Depressive Disorder
Source: PLoS One. 2012 Oct 8;7(10):e46439. doi: 10.1371/journal.pone.0046439 (PMC3466291; doi:10.1371/journal.pone.0046439)
Supplement: Table S2 — Mean (SD) eigenvariates extracted from the peak voxel regions where hemodynamic activity was significantly different between healthy controls and participants with major depressive disorder for masked-sad faces (SN) versus masked-neutral faces (NN). The coordinates correspond to regions reported in Table 3 of the manuscript. (DOCX) [file pone.0046439.s003.docx]

Supplementary Table S2. Mean (SD) eigenvariates extracted from the peak voxel regions where hemodynamic activity was significantly different between healthy controls and participants with major depressive disorder for masked-sad faces (SN) versus masked-neutral faces (NN). The coordinates correspond to regions reported in Table 3 of the manuscript.

| Region | x,y,z | | | Eigenvariate | | | | |
| --- | --- | --- | --- | --- | --- | --- | --- | --- |
|  |  |  |  | SN | | NN | | |
|  |  |  |  | HC | MDD | | HC | MDD |
| Masked-Sad Faces vs. Masked-Neutral Faces, MDD>HC | | | | | | | | |
| L Rostral STG | | | -51, 9, -11 | -0.28 (1.08) | 0.49 (1.28) | | 0.96 (0.92) | -0.18 (1.34) |
| R Rostral STG | | | 51, 0, 0 | -0.32 (0.75) | 0.18 (1.26) | | 0.28 (0.80) | -0.51 (1.07) |
| R Anterior Orbitofrontal C | | | 22, 46, -16 | -0.13 (0.64) | 0.35 (0.92) | | 0.53 (0.87) | -0.13 (0.68) |
| Masked-Sad Faces vs. Masked-Neutral Faces, HC>MDD | | | | | | | | |
| L Inferior Parietal C | | -44, -44, 50 | | 0.67 (0.90) | 0.48 (1.01) | | 0.39 (0.59) | 1.29 (1.00) |
| L Inferior Parietal C | | -49, -38, 46 | | 0.54 (0.82) | 0.36 (0.91) | | 0.36 (0.55) | 1.11 (0.82) |
| R Frontal Polar C | | 34, 60, 4 | | 0.91 (1.01) | -0.61 (1.64) | | 0.06 (1.21) | 0.48 (1.44) |

Abbreviations: SD= standard deviation; SN= masked-sad faces; NN= masked-neutral faces; HC= healthy control; MDD= major depressive disorder; L= left; R= right; STG= superior temporal gyrus; C= cortex
