# Supplementary material for: The Extended Functional Neuroanatomy of Emotional Processing Biases for Masked Faces in Major Depressive Disorder
Source: PLoS One. 2012 Oct 8;7(10):e46439. doi: 10.1371/journal.pone.0046439 (PMC3466291; doi:10.1371/journal.pone.0046439)
Supplement: Table S3 — Mean (SD) eigenvariates extracted from the peak voxel regions where hemodynamic activity was significantly different between healthy controls and participants with major depressive disorder for masked-happy faces (HN) versus masked-neutral faces (NN). The coordinates correspond to regions reported in Table 3 of the manuscript. (DOCX) [file pone.0046439.s004.docx]

Supplementary Table 3. Mean (SD) eigenvariates extracted from the peak voxel regions where hemodynamic activity was significantly different between healthy controls and participants with major depressive disorder for masked-happy faces (HN) versus masked-neutral faces (NN). The coordinates correspond to regions reported in Table 3 of the manuscript.

| Region | x,y,z | | Eigenvariate | | | | |
| --- | --- | --- | --- | --- | --- | --- | --- |
|  |  |  | HN | | NN | | |
|  |  |  | HC | MDD | | HC | MDD |
| Masked-Happy Faces vs. Masked-Neutral Faces, MDD>HC | | | | | | | |
| L Rostral STG | | -53, 4, 2 | -0.08 (0.90) | 0.57 (0.78) | | 0.82 (1.10) | 0.29 (0.77) |
| R Rostral STG | | 51, 15, -2 | 0.05 (1.20) | 0.64 (1.30) | | 1.13 (0.96) | 0.45 (1.36) |
| L Anterior Orbitofrontal C | | -16, 52, -13 | -0.77 (0.99) | -0.07 (0.70) | | 0.04 (0.59) | -0.81 (0.91) |
| L Anterior Insula | | -32, 16, 5 | 0.13 (0.42) | 0.82 (1.01) | | 0.82 (0.74) | 0.37 (0.73) |
| L Pregenual ACC | | -8, 43, 3 | -0.56 (0.70) | 0.04 (0.81) | | -0.31 (0.54) | -0.93 (0.66) |
| R Ventral Thalamus | | 10, -13, 4 | 0.23 (0.62) | 0.66 (0.57) | | 0.72 (0.70) | 0.37 (0.51) |
| R Postcentral G | | 65, -24, 31 | -0.47 (0.97) | 0.14 (0.81) | | 0.26 (0.70) | -0.33 (0.77) |
| Masked-Happy Faces vs. Masked-Neutral Faces, HC>MDD | | | | | | | |
| L Middle Occipital G | | -44, -81, 15 | 0.19 (1.11) | -0.24 (1.24) | | -0.26 (0.92) | 1.00 (1.27) |

Abbreviations: SD= standard deviation; HN= masked-happy faces; NN= masked-neutral faces; HC= healthy control; MDD= major depressive disorder; L= left; R= right; STG= superior temporal gyrus; C= cortex; ACC= anterior cingulate cortex; G= gyrus
